# Dose-enhanced versus standard TTFields for first recurrence of glioblastoma: A randomized phase 2 clinical trial

**DOI:** 10.1093/noajnl/vdaf245

**Published:** 2025-11-20

**Authors:** Nikola Mikic, Slávka Lukacova, Jane Skjøth-Rasmussen, Frantz Rom Poulsen, John Hauerberg, René Johannes Laursen, Kåre Schmidt Ettrup, Søren Møller, Charlotte Aaquist Haslund, Rikke Hedegaard Dahlrot, Søren Ole Stigaard Cortnum, Ann Kathrine Sindby, Gorm Von Oettingen, Jan Brink Valentin, Ole Solheim, Tora Skeidsvoll Solheim, Jens Christian Hedemann Sørensen, Eric T Wong, Anders Rosendal Korshøj

**Affiliations:** Department of Neurosurgery, Aarhus University Hospital; Department of Clinical Medicine, Aarhus University; Department of Clinical Medicine, Aarhus University; Department of Oncology, Aarhus University Hospital; Department of Neurosurgery, Rigshospitalet; Department of Clinical Medicine, Copenhagen University; Department of Neurosurgery, Odense University Hospital; Clinical Institute BRIDGE (Brain Research InterDisciplinary Guided Excellence), University of Southern Denmark; Department of Neurosurgery, Rigshospitalet; Department of Neurosurgery, Aalborg University Hospital; Department of Neurosurgery, Aalborg University Hospital; Department of Oncology, Rigshospitalet; Department of Oncology, Aalborg University Hospital; Department of Oncology, Odense University Hospital; Department of Clinical Research, University of Southern Denmark; Department of Neurosurgery, Aarhus University Hospital; Department of Neurosurgery, Aarhus University Hospital; Department of Neurosurgery, Aarhus University Hospital; Danish Center for Health Services Research, Department of Clinical Medicine, Aalborg University; Department of Neurosurgery, St. Olavs University Hospital; Department of Neuromedicine and Movement Science, Norwegian University of Science and Technology; Department of Oncology, St. Olavs University Hospital; Department of Clinical and Molecular Medicine, Norwegian University of Science and Technology; Department of Neurosurgery, Aarhus University Hospital; Department of Clinical Medicine, Aarhus University; Division of Hematology/Oncology, Department of Medicine, Brown University Health & Rhode Island Hospital, Rhode Island; Department of Neurosurgery, Aarhus University Hospital; Department of Clinical Medicine, Aarhus University

**Keywords:** clinical trial, dose, glioblastoma, TTFields, tumor treating fields

## Abstract

**Abstract:**

BackgroundStudies suggest that high intensities of tumor treating fields (TTFields) correlate with prolonged survival in newly diagnosed glioblastoma. However, no randomized clinical studies have tested different doses of TTFields, and the treatment remains controversial for recurrent glioblastoma. This study examined the clinical efficacy of dose-enhanced TTFields in first recurrence glioblastoma (rGBM).

**Methods:**

This open-label, randomized, multicenter, phase 2 clinical trial was conducted nationwide in Denmark (2020-2024) with planned enrollment of 84 rGBM patients. Inclusion criteria were focal disease, KPS ≥ 70, and eligibility for resection. Patients were randomized (1:1) to receive standard or dose-enhanced TTFields in addition to standard-of-care. Dose enhancement (25%-70%) in the tumor was achieved by placing five small cranial burr holes over the tumor bed with overlapping TTFields transducer arrays. The primary outcome was the overall survival (OS) rate at 12 months (OS12). Secondary outcomes included progression-free survival, toxicity, steroid use, objective response rate (ORR), and quality of life.

**Results:**

We enrolled 58 participants with a mean (SD) age of 59.2 (11.1) years and a median (IQR) KPS of 90 (10). Preplanned interim analysis of the first 52 patients resulted in early trial termination due to futility. Intent-to-treat analysis of the complete cohort showed an OS12 of 56% vs 46% (*P* = .38) and a median OS of 12.3 vs 11.1 months (*P* = .93) for intervention and control, respectively. Differences in the secondary outcomes were insignificant.

**Conclusion:**

Dose-enhanced TTFields utilizing burr holes over the resection cavity were not associated with improved survival in rGBM, with low study power as the primary limitation.

Key Points

- Enhanced TTFields dose (25%-70%) shows no survival benefit in recurrent glioblastoma, contradicting a dose-response relationship.
- This suggests limited benefit of TTFields dose intensification in rGBM, directing research toward other strategies.

Importance of the Study

This randomized trial addresses a critical knowledge gap in neuro-oncology by examining TTFields in 2 doses in glioblastoma. While preclinical studies and observational clinical data suggested that increased TTFields dose improves outcomes, no randomized controlled trials have been conducted to validate these observations. Moreover, controversy remains about the clinical evidence and utility of TTFields in the recurrent setting. Using innovative cranial windows to achieve focal dose enhancement up to 70% in the tumor bed, far exceeding conventional field optimization, we markedly intensified the therapeutic potential of TTFields in recurrent GBM patients. Despite significant dose enhancement, we found no survival improvement. These findings have immediate implications for TTFields optimization strategies, suggesting that optimization of array layout or other dose-maximizing approaches offers limited benefit in recurrent glioblastoma. Our results establish that even optimally delivered TTFields likely cannot extend survival beyond approximately one year in the first recurrence, directing future research toward alternative therapeutic approaches for this devastating disease.

Glioblastoma (GBM) is a deadly cancer without effective treatment for recurrence. Adjuvant tumor treating fields (TTFields) therapy improves overall survival (OS) in newly diagnosed GBM (ndGBM), but its efficacy is less established for recurrent GBM.^1–4^ Observational clinical studies indicate that increased TTFields dose (ie field intensity [V/cm]) improves OS and reduces local recurrence,^5^,^6^ aligning with preclinical results on the dose-response relationship.^7^,^8^ Strategies for improving the TTFields dose have been optimizing transducer array placement with NovoTAL and individualized placement based on tumor pathology, which achieved up to a 20% increase in field dose.^9^ Recently, we developed a surgical method to focally enhance TTFields dose by placing five cranial burr holes over the tumor bed. These low-impedance windows facilitate a 25%-70% increase in the TTFields intensity in the tumor as demonstrated by *in silico* modeling studies.^10–14^ The method’s safety was validated in a phase 1 clinical trial, showing median OS (mOS) of 15.5 months in first recurrence glioblastoma (rGBM),^13^ indicating a favorable efficacy compared to a historical mOS of around 9 months.^15^ This study aimed to further evaluate the clinical efficacy and safety of dose-enhanced TTFields for rGBM in a randomized setting.

## Methods

### Study Design and Participants

In this investigator-initiated, multicenter, open-label, phase 2 randomized clinical trial, we enrolled patients with supratentorial focal rGBM (WHO 2016), KPS ≥ 70, and indication for resection. Patients were excluded if histopathology did not confirm recurrence^16^ (protocol in Document S1). The study was conducted nationwide in Denmark (Copenhagen, Aarhus, Odense, and Aalborg University Hospitals), approved by the Danish Ethics Committee, and followed Good Clinical Practice and ISO-14155 standards. It was monitored by an independent contract research organization and a Data Safety Monitoring Committee (DSMC, Document S2). Centralized independent neuroradiological review and response validation were performed. Reporting followed the CONSORT guidelines. Trial registered at clinicaltrials.gov with identifier: NCT0422399, registered on 13 January 2020.

### Randomization, Masking, and Procedures

Eligible patients were randomized 1:1 to receive dose-enhanced or conventional TTFields in addition to standard-of-care (SOC) treatments. Dose enhancement was achieved with five burr holes (15 mm diameter) in a quincunx shape (4.5 & 4.5 cm) over the resection cavity. TTFields transducer arrays overlapped the burr holes to maximize electric fields in the tumor (Figure 1).^10^ The standard operating procedure can be found in Document S3. Randomization was conducted using block permutation in REDCap and performed perioperatively after resection to avoid surgeon’s bias. The allocation sequence was blinded to all study personnel. Blinding was impossible due to the intervention’s nature. Clinical examination, MRI, quality-of-life (QoL), and toxicity assessment were conducted at inclusion, postoperatively, at TTFields initiation, and every twelve weeks during exposure. Treatment was discontinued upon progression, unacceptable adverse events (AEs), or withdrawal of consent. TTFields beyond progression were allowed for compassionate use.

**Figure 1.** Enhancement of TTFields using cranial burr holes. (A) shows a postoperative 3D CT-C scan with five burr holes, each 1.5 cm in diameter, arranged in a quincunx pattern, and placed over the tumor bed. (B) Shows a computational *in silico* model based on the patient’s postoperative 3D head CT and MRI scans. The five burr holes are shown as blue dots and the TTFields transducer arrays are shown in orange. To maximize field enhancement, the burr holes are placed directly above the tumor with overlapping transducer arrays, represented as 60-degree and 120-degree rotations around a cranio-caudal axis, displaying one array from each of the 2 array pairs, respectively: This position was chosen ensure that an array from each pair overlapped the burr holes to maximize field enhancement. (C) A heat map of the difference in the computed electric dose with and without cranial burr holes (Δ*E* = *E*_burrholes_ − *E*_no burrholes_) presented as surface, axial, sagittal, and coronal views for each array position, respectively. The tumor is visible as a spherical shape on the axial, sagittal, and coronal views. The figure has been used and edited from (10) with permission from the authors.

### Endpoints

The primary endpoint was OS at 12 months (OS12) in the intent-to-treat (ITT) population. Per protocol (PP) analysis was conducted and required ≥4 weeks of active TTFields with ≥75% average compliance. Secondary endpoints included PFS, ORR, QoL (EORTC QLQ-C30 and QLQ-BN20), cumulative corticosteroid dosage, KPS decline, and toxicity (CTCAEv5.0).

### Statistical Analysis

The trial was designed for superiority detection of a 20% increase in OS12 in the intervention group (expected OS12 = 60%), based on two-sided α = 0.15, and 1 − β = 0.80, and an expected OS12 of 40% for control.^15^ Using a two-stage minimax approach, the calculated sample size was 84 patients in total, with 52 in the interim cohort.^17^ Outcomes were analyzed as ITT and PP. Time-to-event data were recorded from inclusion and analyzed using the Kaplan-Meier estimator. Between-group differences were assessed using the log-rank test. Median survival was reported with 95% confidence intervals (CI). We applied Mood’s test of differences between medians of continuous outcomes and the χ^2^ test for categorical outcomes. Analyses were conducted separately by 2 trial-independent biostatisticians in R (R Core Team [2023]).

### Stopping Rules

The trial was designed to stop early in case of futility, ethical, or safety concerns determined by the DSMC. The interim futility criterion was defined as fewer survivors in the intervention group vs control at 12 months follow-up.

## Results

### Interim Analysis

The interim cohort included 52 participants enrolled between 13 November 2020, and 10 July 2023. Due to slow accrual, an early interim analysis was conducted in January 2024 upon the DSMC’s request and indicated futility. ITT analysis showed a numerical 6% increase in OS12 in favor of dose enhancement without statistical significance (*P* = .65), while PP analysis showed no difference (0%, *P* = 0.98). It was concluded that the primary endpoint could not be achieved and that confirmation of the observed 6% OS12 increase would require an additional 250 patients at 80% power. The DSMC recommended trial termination while allowing follow-up completion for enrolled patients (Document S4). Prespecified interim analysis on 10 July 2024, fulfilled the futility criterion, validating the early termination decision.

### Final Analysis

A total of 174 patients were screened for eligibility. The final analysis included 58 patients enrolled between 13 November 2020 and 4 February 2024 (Figure 2). Baseline and treatment characteristics for the ITT population are presented in Table 1 and for the PP population in Table S1. Arms were well-balanced for all major known prognostic factors. All patients were TTFields naïve at inclusion.

**Figure 2.** Patient flow diagram.

**Table 1.** Base and Treatment Characteristics for the final ITT Population (*n* = 58)

	No. (%)^a^		
	Control	Intervention	Overall
Baseline characteristics	(*n* = 30)	(*n* = 28)	(*N* = 58)
Age, mean (SD), years	61.4 (11.7)	56.9 (9.91)	59.2 (11.1)
Male sex	19 (63)	19 (68)	38(66)
Karnofsky performance score, median (Q1, Q3)	90 (90, 100)	90 (90, 100)	90 (90, 100)
Patients on corticosteroids at baseline	14 (47)	16 (57)	30 (52)
Prednisolone equivalent dose, median (Q1, Q3)^b^	50 (42.50, 76.25)	45 (25, 68.75)	50 (25, 75)
MGMT			
Methylated	13 (43)	9 (32)	22 (38)
Unmethylated	17 (56)	19 (68)	36 (62)
IDH1/2			
Wildtype	28 (93)	27 (94)	55 (95)
Mutated^c^	2 (7)	1 (4)	3 (5)
Time to first progression, months, median (Q1, Q3)	9.2 (6.7, 11.8)	7.6 (4.8, 13.1)	7.80 (6.1, 11.6)
Completed concomitant radiotherapy and temozolomide	30 (100)	28 (100)	58 (100)
No. of adjuvant temozolomide cycles, median (Q1, Q3)	5 (4, 6)	5 (2, 6)	5 (2, 6)
**Treatment characteristics**			
Extent of resection based on a postoperative MRI^d^			
Gross total resection	28 (93)	22 (79)	50 (86)
Partial resection	1 (3)	6 (21)	7 (12)
Biopsy	1 (3)	0 (0)	1 (2)
TTFields therapy duration, months, median (Q1, Q3)	1.70 (0.1, 4.6)	3.75 (1, 9.5)	2.80 (0.58, 7.2)
TTFields compliance, % of 24 hours, Median (Q1, Q3)	70 (23.5, 84.3)	64.0 (46.3, 79.3)	69.0 (45.5, 82.0)
Medical oncological treatment^e^			
Temozolomide 200 mg/m2	5 (17)	5 (18)	10 (17)
Lomustine 110 mg/m2	18 (60)	18 (64)	36 (62)
Irinotecan 125 mg/m2 + Bevacizumab 10 mg/kg	4 (13)	6 (21)	10 (17)
Irinotecan 350 mg/m2 + Bevacizumab 10 mg/kg	1 (3)	0 (0)	1 (2)
None	1 (3)	1 (4)	2 (3)
Other	1 (3)	6 (21)	7 (12)

Abbreviations: MGMT, O6-Methylguanine-DNA-methyltransferase; IDH, isocitrate dehydrogenase.

a Percentages may not add up to 100% due to rounding. Brackets indicate % unless otherwise specified.

b Only patients on corticosteroids at baseline were included in this analysis, that is, *n* = 14 for control and *n*  = 16 for intervention.

c All three patients had astrocytoma grade 4 and were included before the WHO2021 classification change.

d Gross total resection was defined as “no residual disease” and “non-measurable disease” on postoperative MRI according to the RANO criteria.

e The medical oncological treatment given from first recurrence until the end of oncological therapy. The same patient may receive several treatments depending on the number of progressions.

#### Efficacy

The ITT analysis showed OS12 of 56% (95% CI, 40%-78%) vs 46% (95% CI, 31%-70%), P = .38, for intervention and control groups, respectively (Figure 3). Correspondingly, the mOS was 12.3 (95% CI, 8.3-19.4) vs 11.1 (95% CI, 9.5-17.6) months, and OS24 was 15% (95% CI, 6%-42%) vs 25% (95% CI, 13%-50%). The survival HR at one-year follow-up was 0.72 (95% CI, 0.3-1.5).

**Figure 3.** Kaplan-Meier curve showing OS of the final intent-to-treat cohort (*n* = 58).

The median PFS was 4.1 (95% CI, 3.4-10.9) vs 3.5 (95% CI, 3-6.5) months with a corresponding PFS6 of 43% (95% CI, 28%-66%) vs 29% (95% CI, 17%-52%) (Figure 4). The collective cohort had an mOS of 12.3 months [95% CI: 9.7-17.4 months] and OS12 was 51% [39-66] (Figure S1).

**Figure 4.** Kaplan-Meier curve showing the progression-free survival for the intent-to-treat population (*n* = 58 patients).

Response assessment revealed that at trial termination, 25 vs 23 patients had progression, 4 vs 3 had stable disease, 1 vs 1 died before progression, and 0 vs 1 had a partial response in the control and intervention groups, respectively. Partial response was observed after a patient had progressed once during the trial and treatment with bevacizumab was initiated.

PP results showed a less pronounced difference regarding survival compared to ITT with an OS12 of 60% (95% CI, 42%-86%) vs 59% (95% CI, 39%-88%), *P* = .91 for intervention and control, respectively, corroborating early termination due to futility (Figure 5). The remaining results were comparable to findings in the ITT population (Document S5).

**Figure 5.** Kaplan-Meier curve showing the OS of the final per protocol population (*n* = 39 patients).

#### Safety and Quality-of-Life

There were no clinically or statistically significant ­differences in the AE risk (Figure S2 and Tables S2-S4), QoL scores (Figures S3 and S4), steroid use (Figure S5), or KPS (Figure S5) between groups. SAEs were causally unassociated with the intervention. Missing data were only observed for QoL and not at random.

## Discussion

Our study is the first to date to test TTFields at different dose levels in a clinical setting. We found that high TTFields enhancement in the tumor did not significantly improve survival in rGBM. This questions the relevance of dose-enhancing measures for rGBM.^9^

While the use of TTFields in rGBM remains controversial following EF-11,^2^ our approach was motivated by the critical need for more effective treatments of this condition and the favorable risk-benefit profile established by OptimalTTF-1.^13^ Moreover, our combination design, incorporating TTFields with standard therapy in both study arms, differed from EF-11's monotherapy approach and was designed to provide a potential added benefit to all participants. This approach is supported by a *post hoc* analysis of EF-14, showing that TTFields combined with chemotherapy significantly improved survival compared to chemotherapy alone in patients with first recurrence of GBM (11.8 vs 9.2 months; P = .049).^18^

Accordingly, our complete cohort had an mOS of 12.3 months and OS12 of 51%. In comparison, the collective cohort of Danish resected rGBM patients between 2016 and 2022 (*n* = 395) has an mOS of 10.2 months (95% CI, 9.3-10.9) and an OS12 of 37% (95% CI, 32-43).^19^ Recent international RANO Resect rGBM data showed an mOS of 11.0 months.^20^ Although these comparative data suggest a potential minor survival benefit in our subjects, which may be associated with adjuvant TTFields exposure, this observation is subject to several confounding factors. Specifically, the absence of a control group without TTFields prevents definitive conclusions regarding the efficacy of TTFields. The observed survival outcomes in both groups may be primarily attributable to stringent patient selection criteria rather than TTFields exposure. Our cohort was enriched with favorable prognostic factors, including good performance status, surgical candidacy, absence of multiple prior recurrences, three patients with IDH-mutated tumors (re-classified as Astrocytoma grade 4 under WHO 2021 criteria), and four patients without histological confirmation of recurrence. Additionally, all patients received contemporary standard medical therapy concurrent with TTFields. These confounding factors collectively may explain the prolonged survival outcomes observed in both study arms, independent of any potential TTFields benefit. Future controlled trials with matched non-TTFields comparator groups will be essential to establish the therapeutic contribution of enhanced TTFields delivery definitively.

Importantly, the procedure for TTFields enhancement was well-tolerated, and no significant toxicity or adverse impact on QoL was observed, indicating that TTFields can be safely used in patients with skull defects.

Considering whether confounding factors may have attenuated the difference in outcomes between the groups in our study, it is important to note that the major prognostic factors were well-balanced. However, it must be considered if the exposure time to TTFields in our subjects was insufficient. Studies have indicated that TTFields treatment takes time to exert its anti-tumor effect. Specifically, a *post hoc* analysis of the EF-11 trial for rGBM indicated that it may take as long as 6 months to achieve tumor control or response.^21^ Furthermore, the local minimum power density (LMiPD in mW/cm^3^), a quantity of power delivered to the gross tumor volume weighted by exposure time,^5^,^12^ was identified to correlate with survival in the EF14 trial for nGBM.^5^ While the subjects in our ITT cohort were maintained on TTFields for a relatively short period, that is, a median exposure time of 3.8 and 1.7 months for intervention and control groups, respectively, the corresponding median exposure times in the PP cohort were 7.1 and 3.6 months, with high compliance rates of 71% and 81%. Thus, despite significant field enhancement and nearly twice the exposure duration in the intervention group vs controls, there was no difference in survival outcomes (mOS = 13.9 vs 13.6 months and OS12 = 60% vs 59%, respectively). This further supports the notion that the cumulative TTFields dose is less important for rGBM and that the dose-response relationship may have reached a plateau at the tested exposure levels.

Another factor to consider is that corticosteroid use may interfere with the efficacy of TTFields therapy. Activation of cGAS/STING inflammosomes and triggering of immunogenic cell death are cellular mechanisms underlying TTFields’ anti-tumor action.^22^ Accordingly, *post hoc* analyses of EF-11 found that prolonged patient survival was associated with low cumulative dexamethasone dose (<4.1 mg) for both intervention and control,^23^ and that the cumulative dose was <10% compared to non-responders.^24^ Our patients used a balanced average of 4.5 dexamethasone equivalents in the interventional group and 5.0 mg in the control group. While this dose range is common for rGBM patients, it may have negated the efficacy of post-surgical TTFields and chemotherapy in both groups.

### Limitations

The open-label design and the high observed screening failure (67%) due to TTFields might have induced bias in our trial. Moreover, early termination reduced the study’s power, and generalization was limited by the fact that one site recruited 60% (35/58) of patients. Determination of the exact individual TTFields exposure would require *post hoc* modeling. Our conclusions pertain to rGBM and do not extrapolate to ndGBM or other cancers. To make any conclusions for ndGBM would require a separate, well-designed randomized clinical trial.

## Conclusion

Our study is the first to evaluate different TTFields dose levels in a randomized setting, concluding that dose-enhanced TTFields did not improve OS in rGBM. This suggests that dose-enhancing measures for TTFields are of limited value in the recurrent setting and warrants investigation of other strategies.

## Supplementary Material

vdaf245_Supplementary_Data

## Data Availability

Anonymized data will be provided upon reasonable request.
