# Supplementary material for: Dose-enhanced versus standard TTFields for first recurrence of glioblastoma: A randomized phase 2 clinical trial
Source: Neurooncol Adv. 2025 Nov 20;7(1):vdaf245. doi: 10.1093/noajnl/vdaf245 (PMC12746598; doi:10.1093/noajnl/vdaf245)

# **Supplementary Material**

Contents

[**Supplementary Material** 1](#_Toc210363020)

[**Supplementary Document 1. Trial Protocol. See separate document.** 2](#_Toc210363021)

[**Supplementary Doccument 2 – Data Safety Monitoring Committee charter** 3](#_Toc210363022)

[**Supplementary Doccument 3 – Standard Operating Procedure for skull remodeling surgery and transducer array placement** 5](#_Toc210363023)

[**Supplementary Doccument 4 – Data Safety Monitoring Committee 5th meeting minutes** 8](#_Toc210363024)

[**Supplementary Table 1 – Base and treatment characteristics for the per-protocol population.** 15](#_Toc210363025)

[**Supplementary Figure 1. Kaplan-Meier curve showing the overall survival of the pooled total cohort (n=58).** 16](#_Toc210363026)

[**Supplementary Document 5. Per-protocol results (n=39)** 17](#_Toc210363027)

[**Supplementary Figure 2. Swimmer’s plot for the intent-to-treat population (n=58) that shows all patients' deaths, progressions, and SAE.** 18](#_Toc210363028)

[**Supplementary Table 2. A summary of all adverse events graded by the Common Terminology Criteria for Adverse Events v5.** 19](#_Toc210363029)

[**Supplementary Table 3. Overview of AEs grade 1-2.** 20](#_Toc210363030)

[**Supplementary Table 4. Overview of all SAEs (CTCAEv5 grades 3-5).** 21](#_Toc210363031)

[**Supplementary Figure 3. QLQ-C30 data for the intent-to-treat population** 22](#_Toc210363032)

[**Supplementary Figure 4. QLQ-BN20 for the intent-to-treat population** 25](#_Toc210363033)

[**Supplementary Figure 5. Prednisolone equivalent dose and KPS for the ITT population** 27](#_Toc210363034)

## **Supplementary Document 1. Trial Protocol. See separate document.**

**Supplementary Doccument 2 – Data Safety Monitoring Committee charter**

**A summary of the Data Monitoring Committee Charter (DSMC), including the members and responsibilities. Version 2.0, dated 7.11.2020.**

**DSMC Membership**

The DSMC will be composed of a chairperson (neuro-oncologist) and 5 additional voting members for a committee consisting of 3 clinicians (neuro-oncologist, neurosurgeon, nurse), 1 biostatistician and 2 public representatives (one from the USA and one from Denmark). Additional members may be added as required to facilitate the functioning of the DSMC. A DSMC Coordinator shall provide the logistical management and support of the DSMC but is not a member of the DSMC.

The Protocol Statistician will ensure that planned data review materials are delivered to the DSMC, facilitating the closed session discussion, distributing restricted-access video conference information before each meeting, producing DSMC ad hoc requests as needed, and the open and closed session meeting minutes. The Protocol Statistician is not a committee member and does not vote but will be the main point of contact concerning data and analysis-related materials.

**Responsibilities of the DSMC**

The initial responsibility of the DSMC will be to review the safety data package at planned intervals during the trial. The overall DSMC responsibilities are to:

- Make recommendations on further study conduct. Continuation, termination, or other modifications of the study should be based on observed beneficial or adverse effects of any treatment(s) under study or low probability of achieving study objectives.
- Consider essential parts of the study (e.g. protocol adherence, subject withdrawals, protocol violation/deviations) that might be early indicators that may impact safety;
- Make recommendations about study conduct, enrollment, and sample size and/or data collection;
- Perform a risk assessment to weigh possible safety disadvantages;
- Evaluate the Safety Management Plan and protocol in advance of the start of the studies and during the study;
- Make recommendations as to whether the trial will continue as initially designed, be modified, or be terminated;
- Consider factors external to the study when relevant information becomes available, such as scientific or therapeutic developments and/or including comments from the IRB/IEC that approve, disapprove, or alter the protocol, that may have an impact on the safety of the participants, the ethics of the study, or the need to continue the study; and
- Maintain the confidentiality of the study data and results. It is important to note that Aarhus University Hospital decides to terminate or substantially alter a study, although the DSMC recommended actions are considered.

**Meetings**

Before officially convening to discuss the study, data, and monitor safety, the DSMC will have an initial Organizational Meeting. The DSMC will review the progress of the study and cumulative safety data on a periodic basis at twice-yearly review meetings via videoconferencing. The DSMC may make suggestions to add to or reduce the frequency of review meetings.

**Data Monitoring Committee Members and Support Staff**

| **Name** | **Role** | **Institution** |
| --- | --- | --- |
| Nicholas A Butowski, MD | Chair of DSMC (Neuro-Oncologist) | Department of Neurosurgery, University of California, San Francisco, Box 0372, 400 Parnassus Avenue, UC Clinics, A808, San Francisco, CA 94143, USA |
| Zhong-ping Chen, MD, PhD | DSMC Member  (Neurosurgeon) | Department of Neurosurgery, Sun Yat-sen University Cancer Center, 651 Dongfeng Road East, Guangzhou 510060, P.R.China |
| Lee-Jen Wei, PhD | DSMC Member (Biostatistician) | Department of Biostatistics, Harvard School of Public Health, 677 Huntington Avenue, Boston, MA 02115, USA |
| Mary Ann Laubacher, RN, BSN, MN | DSMC Member  (Nurse) | Musella Foundation for Brain Tumor Research & Information, 1100 Peninsula Boulevard, Hewlett, NY 11557, USA |
| Amy D Westermann, MPH, CHES | DSMC Member (Public Representative) | Musella Foundation for Brain Tumor Research & Information, 1100 Peninsula Boulevard  Hewlett, NY 11557, USA |
| Hanne Lisby | DSMC Member (Public Representative) | Brain Tumor Association, Rebildparken 69, 9220, Aalborg Ø, Denmark |
| Bo Martin Bibby, PhD | Protocol Statistician | Department of Public Health - Biostatistical Advisory Service, Aarhus University Bartholins Allé 2, Aarhus C, 8200, Denmark |
| Nikola Mikic, MD, PhD-fellow | DSMC Coordinator | Department of Neurosurgery, Aarhus University Hospital, Palle Juul-Jensens Boulevard 165, Aarhus N, 8200, Denmark |

## **Supplementary Doccument 3 – Standard Operating Procedure for skull remodeling surgery and transducer array placement**

Note: The original SOP was written in Danish and has been translated to English for publication.

**Purpose**

The standard operating procedure for SR-surgery aims to ensure reproducibility and standardization across centers in the trial.

**Randomization**

Randomization is done after the resection is complete. If the participant is randomized to “intervention,” SR-surgery should be performed according to this SOP.

**Configuration and size**

SR-surgery consists of five burr holes, each 15mm in diameter, in a quincunx configuration using a custom drilling template (Figure 1). There are no requirements regarding the surgical tools used to create the burr holes. These may be selected based on the surgeon's preference and typically include high-speed drills or a perforator.

**Location**

The burr holes should be placed directly above the resection cavity. This may be confined to surgical craniotomy, although this is not a requirement. In rare cases, it might be necessary to place the burr holes entirely outside of the bone flap to ensure they are directly above the resection cavity, as shown in Figure 1B. Surgical challenges regarding the placement of burr holes should be discussed with the trial sponsor before surgery to ensure uniformity across the trial.


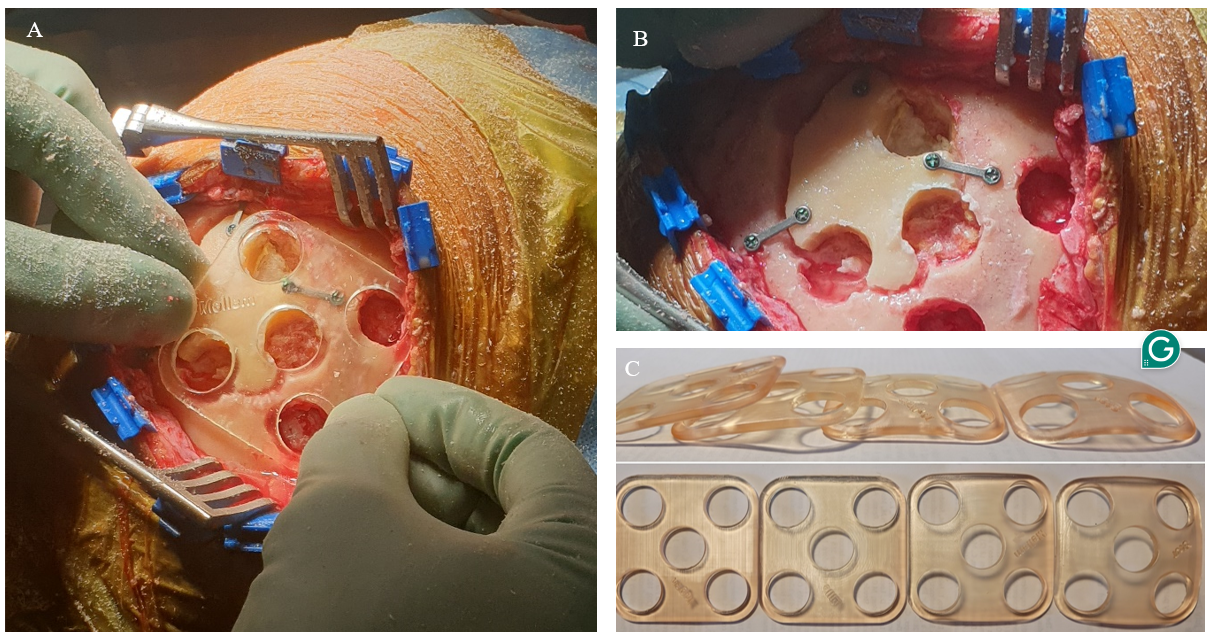


**Figure 1. SR-surgery configuration.** A sterile 3D template is provided for intraoperative use for each patient to ensure reproducibility. Each template is slightly more curved (C), and the closest fit of the skull's curvature should be used. Drilling should not be performed with the template on the bone. Instead, it should be used as visual guidance (A). The template should not be used as an implant, and it should be discarded after use. The size of the burr holes should be checked continuously during the procedure to ensure correct configuration (B).

**Transducer array placement**

Each transducer array layout depends on the SR-surgery location. Arrays should be placed so electrodes from each array pair overlap the burr holes. The concept is illustrated in Figure 2. Each trial participant should be discussed with the Sponsor to ensure the transducer arrays are positioned to optimize the field intensity according to the in silico studies

Further details can be found in Cao et al, 2022 (6).


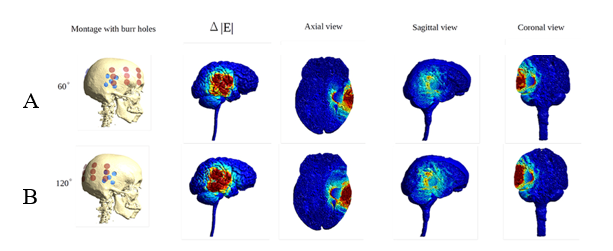
**Figure 2. TTFields array positioning.** Transducer arrays should be placed so that one array from each pair overlaps the burr holes as illustrated in the top row (pair A) and the bottom row (pair B). For each illustrated position the other array in the pair is located opposite on the contralateral side of the head. The figure illustrates the highly focused field enhancement that is achieved with this configuration.

## **Supplementary Doccument 4 – Data Safety Monitoring Committee 5th meeting minutes**

The 5th DSMC meeting was held on January 16th, 2024. At the DSMC's request, an early interim analysis was performed on the first 52 patients included, but six patients lacked the complete 12-month follow-up. The data was presented and discussed, and it was concluded that there was no clinically meaningful benefit from the intervention and that the trial should terminate after completing the follow-up of the 52 patients.

Note: The data are presented as they were at the meeting without modification.

| Protocol: OptimalTTF-2 Phase 2 |
| --- |
| Meeting Date: 18^th^ January 2024 |
| Meeting Attendees (Voting Membership Only): |
| 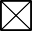 Nicholas A Butowski, MD – DMC Chairperson |
| 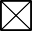 Zhong-ping Chen, MD – DMC Member |
| 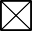 Lee-Jen Wei, PhD – DMC Member |
| 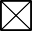 Mary Ann Laubacher, RN, BSN, MN – DMC Member |
| 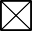 Amy D Westermann, MPH, CHES – DMC Member |
| 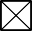 Hanne Lisby – DMC member |

**Absent:** BMB

**Present other:** ARK, ETW,NM

Open and unblinded session

**NM:**

The attached files shown below were presented.

- KM curves for ITT and PP (Figures 1 and 2, respectively.
- Detailed statistical analysis for ITT and PP (Tables 1 and 2, respectively)
- Conditional power calculation for 20% increase in 12OS based on the current trend.

**DSMC:**

It was then discussed that the trend was towards a 3-month overall survival benefit for the intervention arm and a 6% increase in 12OS; however, it would require approximately 250 or 330 patients in total to statistically confirm this trend, assuming 80 and 90% power respectively.

It would not be possible to achieve a 20% increase in the 12OS between the two arms, but if we considered a 6% increase clinically meaningful, we could do a futility analysis to determine the required number of patients and whether continuing the trial was meaningful.

It was concluded that 6% was not enough to justify SR surgery. Furthermore, two other issues were highlighted logistical and ethical concerns. First, the overall recruitment is slow since it appears the other sites have lost interest. Second, it is hard to justify recruiting when the hypothesized significant effect of SR-surgery is not possible to achieve with the current data and trend; it would likely take years to finish the intended recruitment of 84 patients, including the follow-up, making the data less trustworthy while continuing to expose patients to an experimental surgical treatment.

It was concluded that because of the data and the logistical and ethical concerns, we should stop actively recruiting patients and keep the planned follow-up of the 52 first patients. The trial should be terminated once the one-year follow-up of the last 52 patients has been completed.

Enhancing Tumor Treating Fields Therapy for Recurrent Glioblastoma with Targeted and Individualized Skull Remodeling Surgery. A Multi-Center, Randomized Phase 2 Trial.

Our DMC recommendation for this trial is:


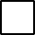
 Continuation of study per protocol without change

Make a modification to study conduct or the protocol, as specified in the comments


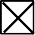


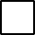
 Termination of study due to safety concerns, as specified in the comments


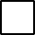
 A further meeting is required to discuss analyses not available today. The additional analyses are specified in the comments.

Comments:

Close for active enrollment. Terminate the trial after the follow-up of the first 52 patients is complete.

Date: 29 Jan 2024


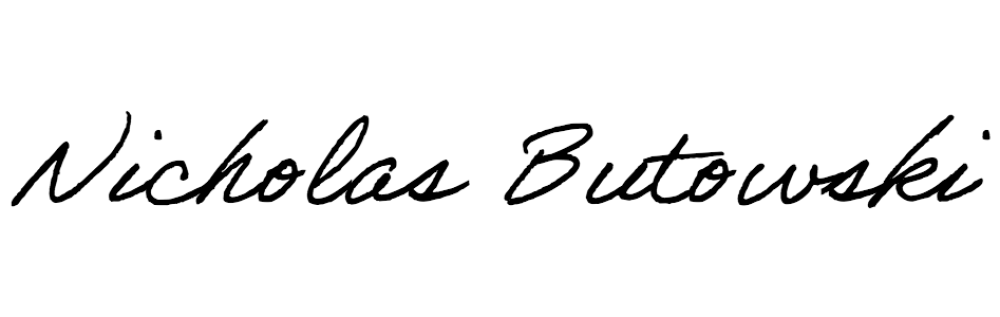
**DMC Chairperson Signature Nicholas A Butowski, MD**

**Table 1. Intent-to-treat analysis of the first 52 patients included in the trial.**

Six patients lacked the complete 12-months follow-up.


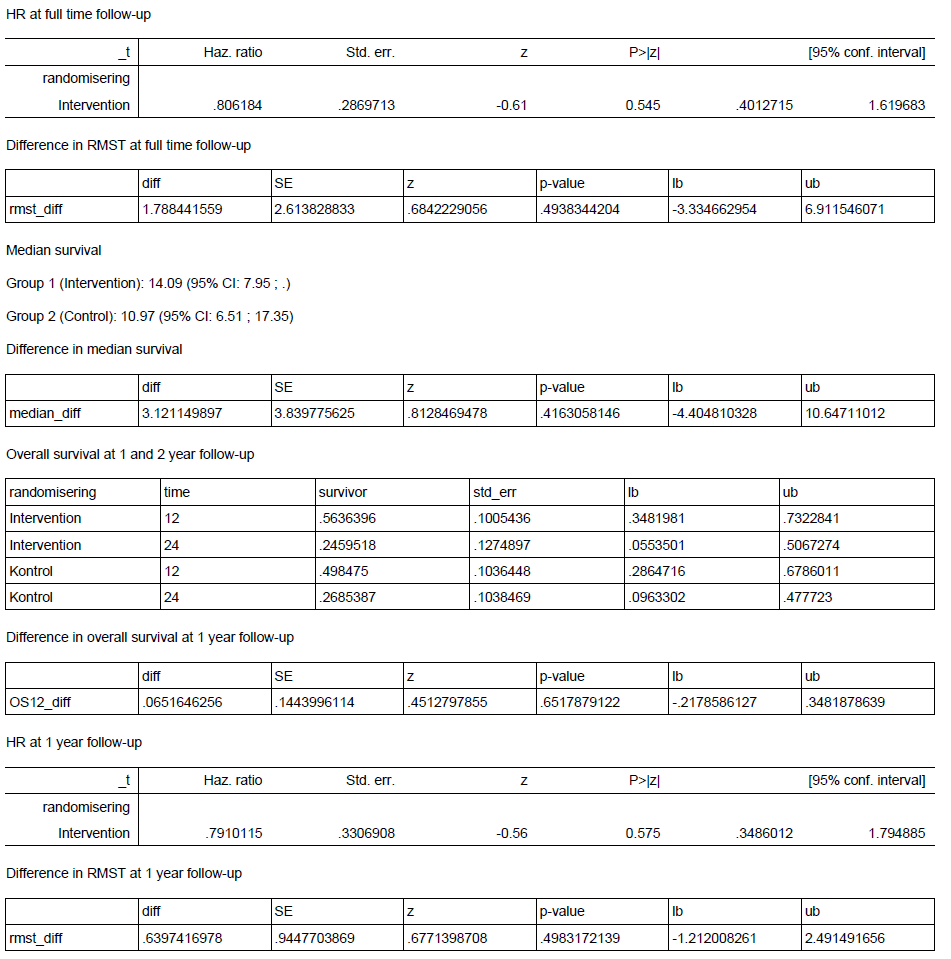


**Figure 1. Kaplan-Meier curve for the intent-to-treat population.** The ITT population included 52 patients of which six lacked complete 12-month follow-up**.**


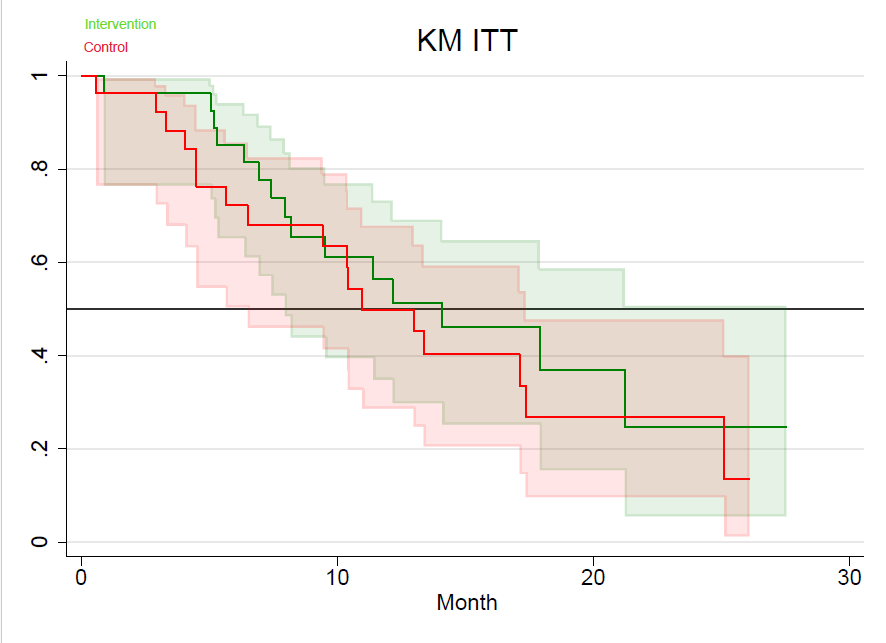


**Table 2. Per-protocol analysis of the first 52 patients included in the trial.**

Six patients lacked complete 12-months follow-up.


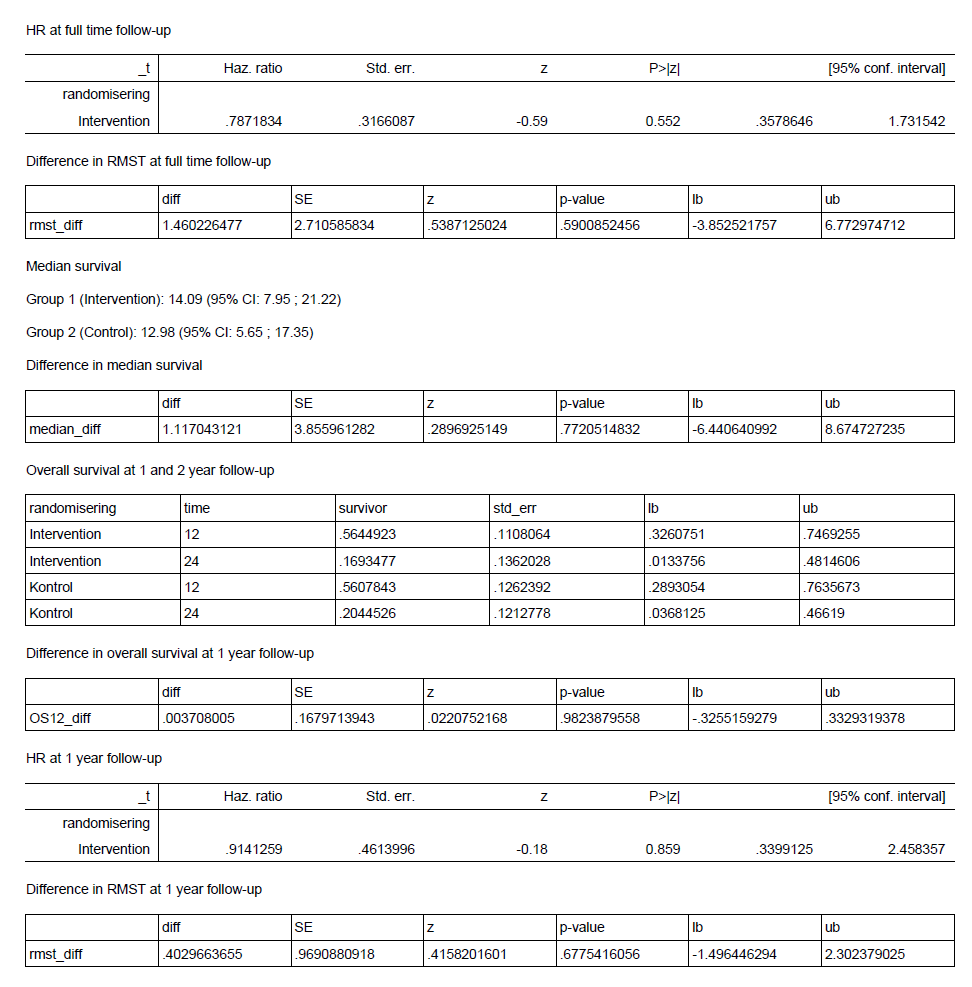


**Figure 2. Kaplan-Meier curve for the per-protocol population of 52 patients.**

Six patients lacked complete 12-month follow-up.


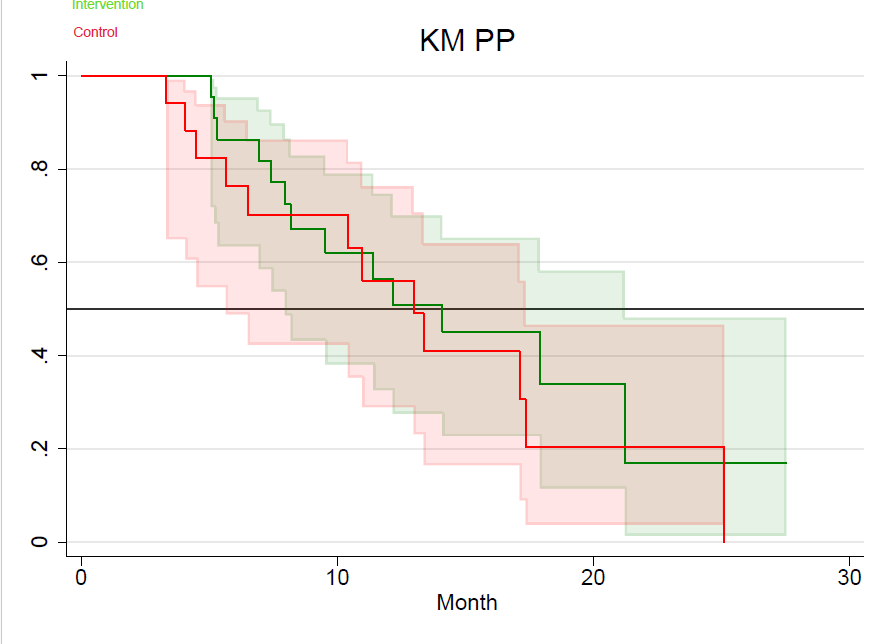


## **Supplementary Table 1 – Base and treatment characteristics for the per-protocol population.**

|  | **No. (%)^a^** | | |
| --- | --- | --- | --- |
|  | **Control** | **Intervention** | **Overall** |
| **Baseline Characteristics** | **(n=18)** | **(n=21)** | **(N=39)** |
| Age, mean (SD), years | 58.9 (12.2) | 55.5 (10.3) | 57.1 (11.3) |
| Male sex | 9 (50) | 17 (81) | 26 (67) |
| Karnofsky performance score, median (Q1,Q3) | 95 (90,100) | 100 (90,100) | 100 (90,100) |
| Patients on corticosteroids at baseline | 8 (44) | 10 (48) | 18 (46) |
| Prednisolone equivalent dose, mg, median (Q1,Q3)^b^ | 62,50 (24.5,75) | 50 (36.25,77) | 50 (36.25,75.25) |
| MGMT |  |  |  |
| Methylated | 7 (39) | 7 (33) | 14 (36) |
| Unmethylated | 11 (61) | 14 (67) | 25 (6) |
| IDH1/2 |  |  |  |
| Wildtype | 17 (94) | 20 (95) | 37 (95) |
| Mutated^c^ | 1 (6) | 1 (5) | 2 (5) |
| Time to first progression, months, median (Q1,Q3) | 10.2 (6.8,12.8) | 7.8 (6.5,14.2) | 8.9 (6.8,13.8) |
| Completed concomitant radiotherapy and temozolomide | 18 (100) | 21 (100) | 39 (100) |
| No. of adjuvant temozolomide cycles, median (Q1, Q3) | 2 (1,3) | 2 (2,3) | 4 (2,6) |
| **Treatment Characteristics** |  |  |  |
| Extent of resection based on postoperative MRI^d^ |  |  |  |
| Gross total resection | 16 (89) | 18 (86) | 34 (87) |
| Partiel resection | 1 (6) | 3 (14) | 4 (10) |
| Biopsy | 1 (6) | 0 (0) | 1 (2) |
| TTFields therapy duration, months, median (Q1,Q3) | 3.6 (2.1,6.8) | 7.1 (3.3,10.6) | 4.60 (2.6,9.6) |
| TTFields compliance, % of 24 hours, median (Q1,Q3) | 81.5 (62.6,86.2) | 71 (60,81) | 75 (61.5,85) |
| Medical oncological treatment^e^ |  |  |  |
| Temozolomide 200 mg/m2 | 5 (28) | 4 (19) | 9 (23) |
| Lomustine 110 mg/m2 | 14 (78) | 15 (71) | 29 (74) |
| Irinotecan 125 mg/m2 + Bevacizumab 10 mg/kg | 4 (22) | 6 (28) | 10 (26) |
| None | 1 (6) | 1 (5) | 2 (5) |
| Other | 1 (6) | 6 (29) | 7 (18) |
|  |  |  |  |

^a^Percentages may not add up to 100% due to rounding. Brackets indicate % unless otherwise specified.

^b^Only patients on corticosteroids at baseline were included in this analysis, i.e. n=8 for control and n=10 for intervention.

^c^Both patients had astrocytoma grade 4 and were included before the WHO2021 classification change.

^d^Gross total resection was defined as “no residual disease” and “non-measurable disease” on post-operative MRI according to the RANO criteria

^e^The same patient may receive several treatments depending on the number of progressions.

O6-Methylguanine-DNA-methyltransferase (MGMT)

Isocitrate dehydrogenase (IDH)

## **Supplementary Figure 1. Kaplan-Meier curve showing the overall survival of the pooled total cohort (n=58).**

The overall survival rate at 12 months was 51% (95%CI, 39-66).


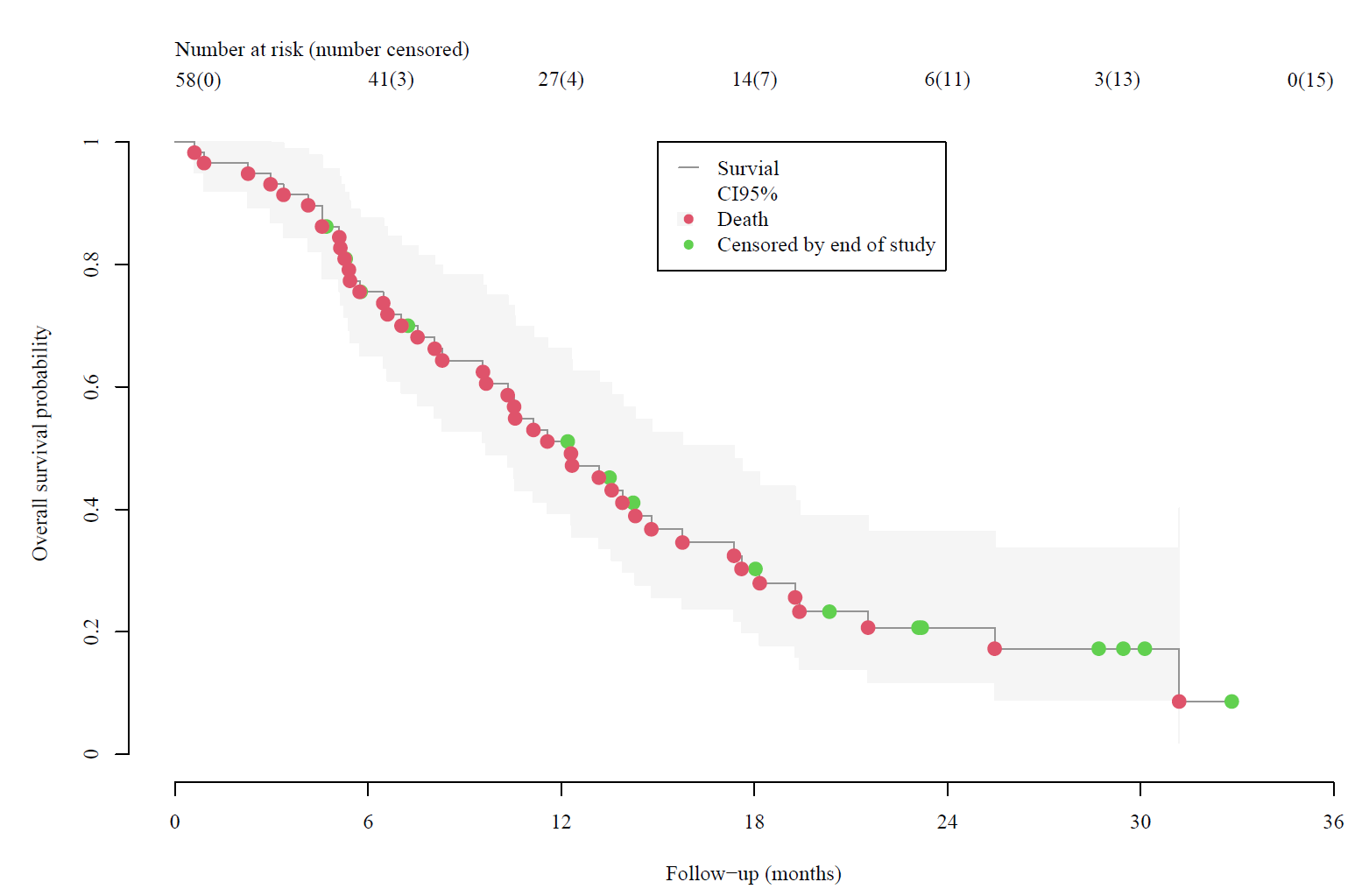


## **Supplementary Document 5. Per-protocol results (n=39)**

PP analysis showed an OS12 of 60% (95% CI, 42-86%) vs. 59% (95% CI, 39-88%), P =.91 for intervention and control, respectively, corroborating early termination for futility. Correspondingly, OS24 was 11% (95% CI, 3-41%) vs. 22% (95% CI, 8-59%), and mOS was 13.9 (95% CI, 9.7-19.4) vs. 13.6 (95% CI, 6.6-50) months, respectively. PFS was 4.6 (95% CI, 3.4-10.9) vs 3.7 (95% CI, 3.3-9.3) months and PFS6 was 43% (95% CI, 26-70%) vs. 32% (95%CI, 16-64%). The HR at one-year follow-up was 0.79 (95% CI, 0.2-2.2). Only one patient in the intervention group had a partial response but was also treated with Bevacizumab. There were no clinically or statistically significant differences in the AE risk, QoL scores, steroid use and KPS between groups. SAEs were causally unassociated with the intervention. QoL results were comparable to previous studies.

**Supplementary Figure 2. Swimmer’s plot for the intent-to-treat population (n=58) that shows all patients' deaths, progressions, and SAE.**


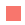
 Death.
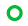
 Progression.
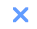
 SAE.


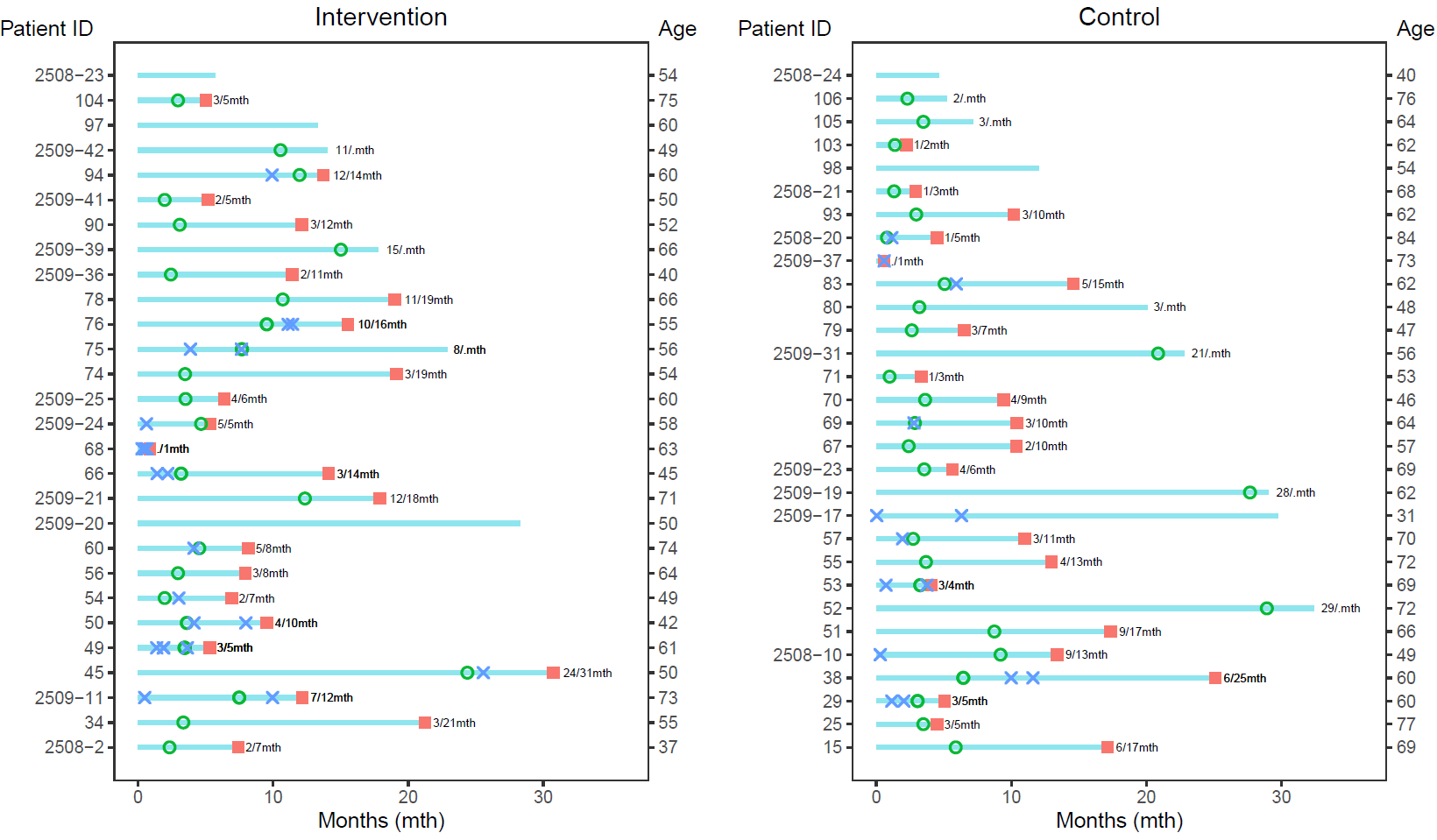


**Supplementary Table 2. A summary of all adverse events graded by the Common Terminology Criteria for Adverse Events v5.**

The independent Data Monitoring Committee evaluated all serious adverse events and did not find plausible causation, concluding that the intervention was safe. The percentage may not add up to 100% due to rounding. The P value was calculated using Fischer’s exact test.

|  | | | | |  |
| --- | --- | --- | --- | --- | --- |
|  |  | **Control** | **Intervention** | **Overall** | **P value** |
|  |  | (N=30) | (N=28) | (N=58) |  |
|  |  |  |  |  |  |
| All SAEs |  | 13 | 22 | 34 | .20 |
| Grade 5^a)^ |  | 1 | 1 | 2 | >.99 |
| Patients with ≥ 1 occurrence, no. (%) |  | 1(3) | 1(4) | 2(3) | >.99 |
| Related to intervention |  | 0 | 0 | 0 | NA^b)^ |
| Grade 4 |  | 0 | 0 | 0 | NA |
| Grade 3 |  | 12 | 21 | 33 | .19 |
| Patients with ≥1 occurrence, no (%) |  | 10 (33) | 12 (43) | 22 (38) | >.80 |
| Related to intervention |  | 0 | 0 | 0 | NA |
| SAEs leading to TTFields discontinuation |  | 0 | 0 | 0 | NA |
| Grade 1-2 |  | 90 | 103 | 193 | 0.55 |
| Patients with ≥1 occurrence,no(%) |  | 24 (80) | 24 (86) | 48 (82) | >.99 |

^a^Pulmonary embolism in both cases

^b^Not applicable.

## **Supplementary Table 3. Overview of AEs grade 1-2.**

The independent Data Monitoring Committee evaluated all AEs and found no statistically or clinically significant difference in toxicity between groups. Due to rounding, percentages may not add up to 100%. The P value was calculated using Fischer’s exact test.

a) Resulting back/shoulder pain

|  | **Control** | **Intervention** | **Overall** | **P value** |
| --- | --- | --- | --- | --- |
|  | (N=30) | (N=28) | (N=58) |  |
| Skin reaction |  |  |  |  |
| Total AE occurrences, no. | 21 | 28 | 49 | .43 |
| Patients with AE, no.(%) | 10 (33) | 13 (46) | 23 (40) | .62 |
| Fatigue |  |  |  |  |
| Total AE occurrences, no. | 18 | 29 | 47 | .23 |
| Patients with AE, no.(%) | 14 (47) | 16 (53) | 30 (52) | .82 |
| Headache |  |  |  |  |
| Total AE occurrences, no. | 24 | 23 | 46 | >.99 |
| Patients with AE, no.(%) | 11 (37) | 16 (57) | 27 (47) | .36 |
| Focal seizure |  |  |  |  |
| Total AE occurrences, no. | 8 | 11 | 19 | .59 |
| Patients with AE, no.(%) | 3 (10) | 7 (25) | 9 (16) | .31 |
| Subcutaneous CSF collection |  |  |  |  |
| Total AE occurrences, no. | 4 | 4 | 8 | >.99 |
| Patients with AE, no.(%) | 4(13) | 4(14) | 8 (14) | >.99 |
| Equipment burden^a)^ |  |  |  |  |
| Total AE occurrences, no. | 6 | 1 | 7 | .12 |
| Patients with AE, no.(%) | 4 (13) | 1 (4) | 5 (9) | .36 |
| Fever |  |  |  |  |
| Total AE occurrences, no. | 4 | 2 | 6 | .67 |
| Patients with AE, no.(%) | 2 (7) | 1 (4) | 3 (5) | >.99 |
| Thromboembolic events |  |  |  |  |
| Total AE occurrences, no. | 3 | 1 | 4 | .61 |
| Patients with AE, no.(%) | 2 (7) | 1 (4) | 3 (5) | >.99 |
| Generalized seizures |  |  |  |  |
| Total AE occurrences, no. | 1 | 1 | 2 | >.99 |
| Patients with AE, no.(%) | 1 (3) | 1 (4) | 2 (3) | >.99 |
| Wound dehiscence |  |  |  |  |
| Total AE occurrences, no. | 1 | 1 | 2 | >.99 |
| Patients with AE, no.(%) | 1 (3) | 1 (4) | 2 (3) | >.99 |
| Surgical wound infection |  |  |  |  |
| Total AE occurrences, no. | 0 | 1 | 1 | >.99 |
| Patients with AE, no.(%) | 0 | 1 (4) | 1 (2) | >.99 |

**Supplementary Table 4. Overview of all SAEs (CTCAEv5 grades 3-5).**

The independent DSMC evaluated all SAEs and found no plausible causation, concluding that the intervention was safe. Due to rounding, the percentage may not add up to 100%. The P value was calculated using Fischer’s exact test.

|  | **Control** | **Intervention** | **Overall** | **P value** |
| --- | --- | --- | --- | --- |
|  | (N=30) | (N=28) | (N=58) |  |
| Grade 5, no. | 1 | 1 | 2 | >.99 |
| Pulmonary embolism, no. (%) | 1 (3) | 1 (4) | 2 (3) | >.99 |
| Grade 4, no. | 0 | 0 | 0 | NA |
| Grade  3, no. | 12 | 21 | 33 | .19 |
| Patients with grade 3 AE, no.(%) | 10 (33) | 12 (43) | 22 (38) | >.80 |
| Generalized seizure, no. (%) | 2 (7) | 4 (14) | 6 (10) | .67 |
| Patients with generalized seizure, no.(%) | 2 (7) | 2 (7) | 4 (7) | >.99 |
| Focal seizure, no. (%) | 1 (3) | 5 (18) | 6 (10) | .20 |
| Patients with focal seizure, no.(%) | 1 (3) | 3 (11) | 4 (7) | .61 |
| Urosepsis, no. (%) | 1 (3) | 3 (11) | 4 (7) | .61 |
| Patients with urosepsis, no.(%) | 1 (3) | 3 (11) | 4 (7) | .61 |
| Headache, no. (%) | 0 (0) | 2 (7) | 2 (3) | .49 |
| Patients with headache, no.(%) | 0 (0) | 1 (4) | 1 (2) | >.99 |
| Infection, unspecified, no. (%) | 0 (0) | 2 (7) | 2 (3) | .49 |
| Patients with unspecific infection, no.(%) | 0 (0) | 2 (7) | 2 (3) | .49 |
| Pulmonary embolism, no. (%) | 2 (7) | 0 (0) | 2 (3) | .49 |
| Patients with pulmonary embolism no. (%) | 2 (7) | 0 (0) | 2 (3) | .49 |
| Kidney stone, no. (%) | 0 (0) | 1 (4) | 1 (2) | >.99 |
| Wound dehiscence, no. (%) | 0 (0) | 1 (4) | 1 (2) | >.99 |
| Stroke, MCA, no. (%) | 0 (0) | 1 (4) | 1 (2) | >.99 |
| Personality change, no. (%) | 1 (3) | 0 (0) | 1 (2) | >.99 |
| Lymfocytopenia, no. (%) | 0 (0) | 1 (4) | 1 (2) | >.99 |
| Pneumonia, no. (%) | 1 (3) | 0 (0) | 1 (2) | >.99 |
| Abdominal pain, no. (%) | 1 (3) | 0 (0) | 1 (2) | >.99 |
| Cerebral edema, no. (%) | 1 (3) | 0 (0) | 1 (2) | >.99 |
| Syncopy, no (%) | 1 (3) | 0 (0) | 1 (2) | >.99 |
| Cerebral venous sinus thrombosis, no. (%) | 1 (3) | 0 (0) | 1 (2) | >.99 |

**Supplementary Figure 3. QLQ-C30 data for the intent-to-treat population.**

T0 = Baseline, T1 = Period postsurgery but before TTFields, T2 = 3-month control, T3 = 6-month control. The scores were calculated following the standardized recommended EORTC approach. Mean values are shown with 95% confidence interval. While there are clinically meaningful changes in the values over time in both arms, these are attributed to glioblastoma disease, its treatment, and progression. There was no worsening in the dose-enhanced arm that could be attributed to the intervention. Here the global health status and the five functional domains, physical, role, emotional, cognitive, and social are shown. The higher the score the better the function.
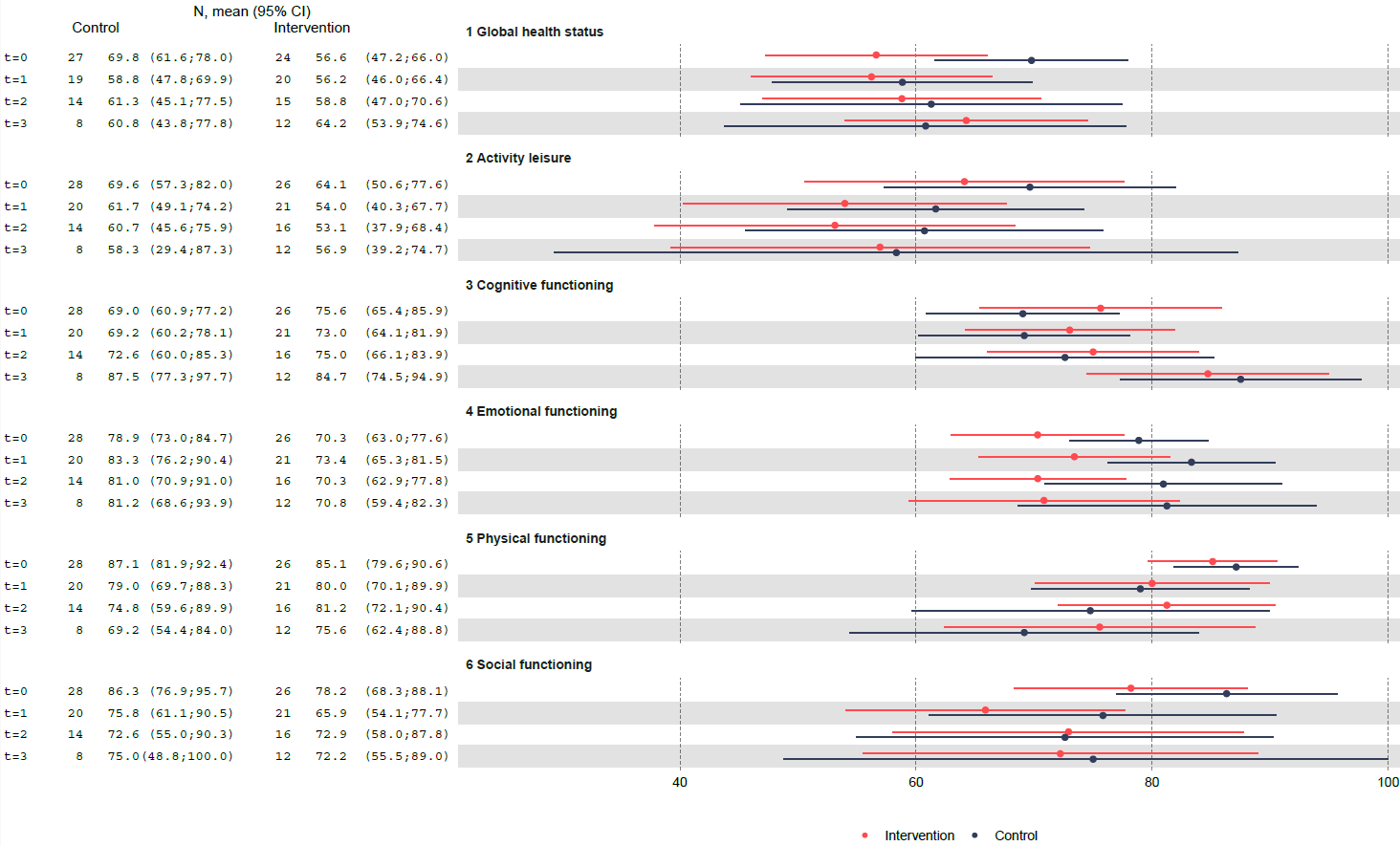


**Supplementary Figure 3. QLQ-C30 data for the intent-to-treat population (continued).**

T0 = Baseline, T1 = Period postsurgery but before TTFields, T2 = 3-month control, T3 = 6-month control. The scores were calculated following the standardized recommended EORTC approach. Mean values are shown with 95% confidence interval. While there are clinically meaningful changes in the values over time in both arms, these are attributed to glioblastoma disease, its treatment, and progression. There was no worsening in the dose-enhanced arm that could be attributed to the intervention. The symptom score shown is worse the higher the score is.
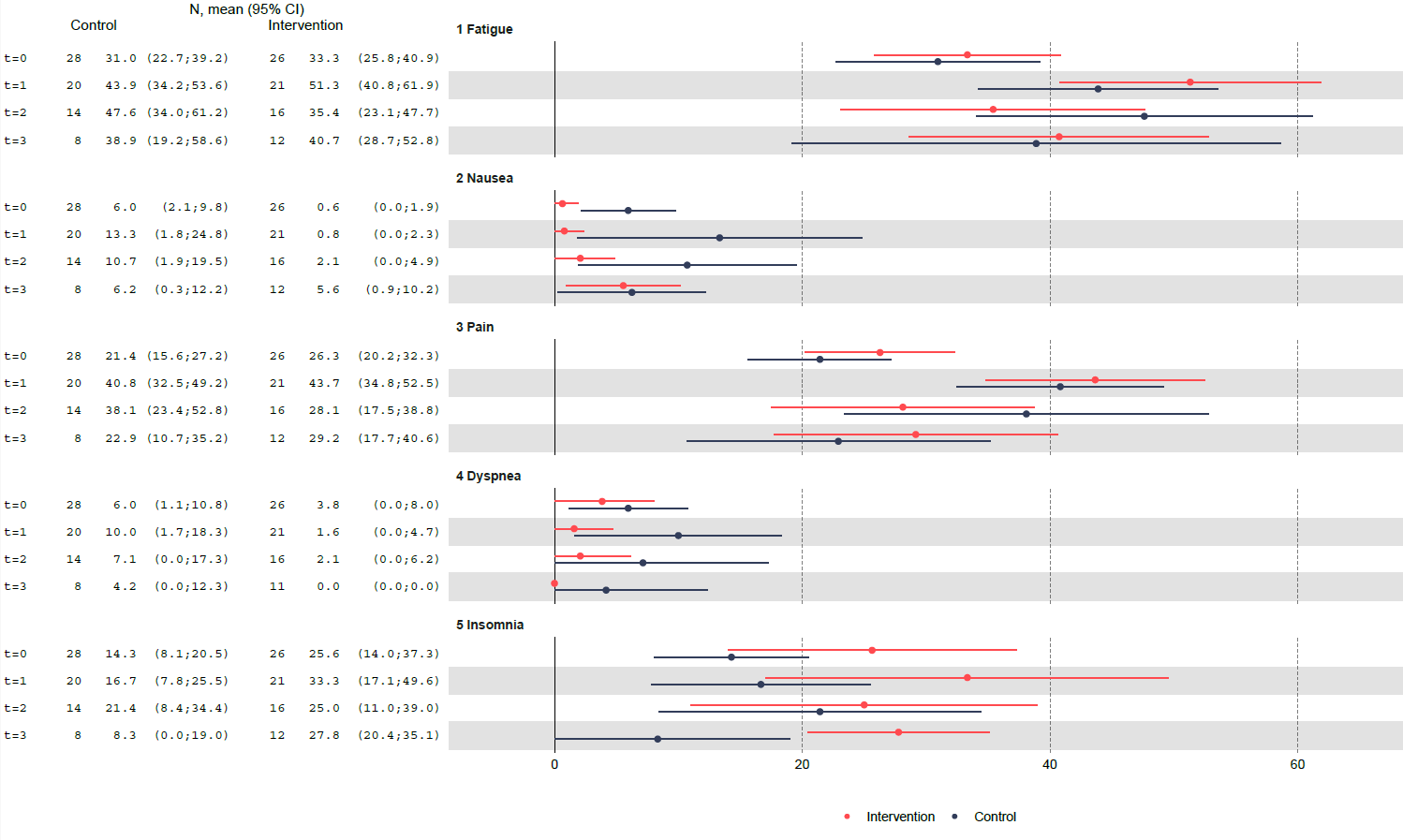


**Supplementary Figure 3. QLQ-C30 data for the intent-to-treat population (continued).**

T0 = Baseline, T1 = Period postsurgery but before TTFields, T2 = 3-month control, T3 = 6-month control. The scores were calculated following the standardized recommended EORTC approach. Mean values are shown with 95% confidence interval. While there are clinically meaningful changes in the values over time in both arms, these are attributed to glioblastoma disease, its treatment, and progression. There was no worsening in the dose-enhanced arm that could be attributed to the intervention, such as headache or itchy skin. The symptoms score and financial difficulty are worse the higher the score.
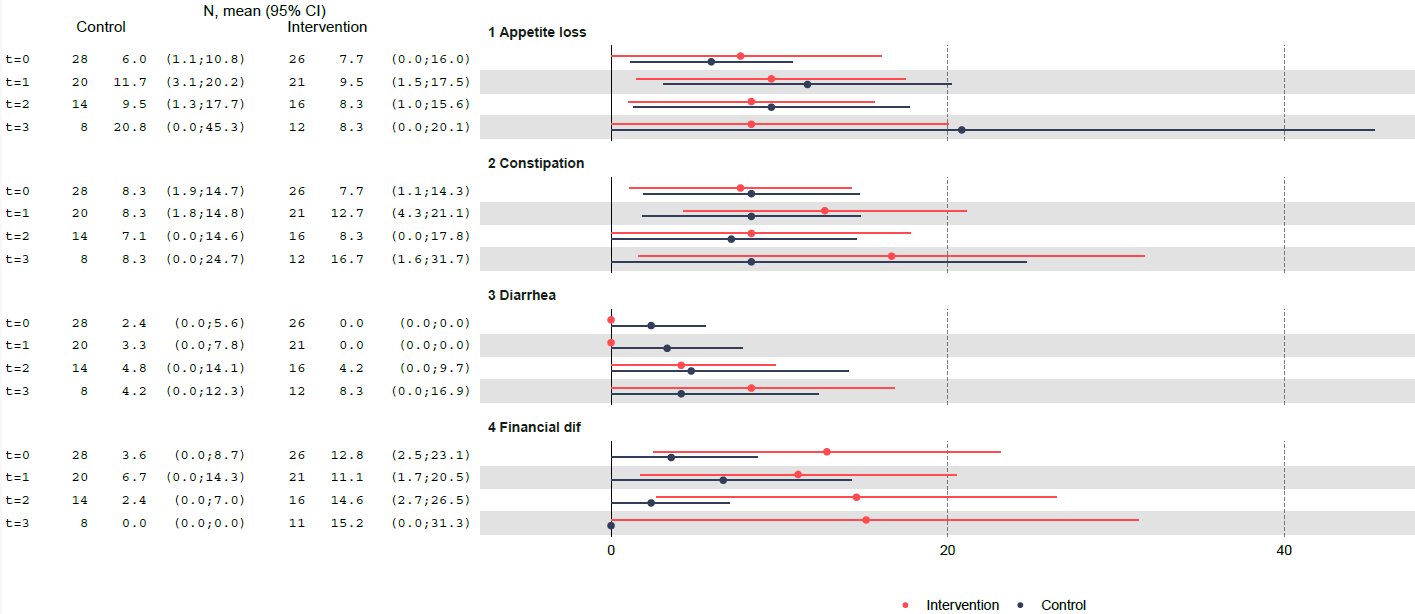


**Supplementary Figure 4. QLQ-BN20 for the intent-to-treat population.**

T0 = Baseline, T1 = Period postsurgery but before initiating TTFields, T2 = 3-month control, T3 = 6-month control. The scores were calculated following the standardized recommended EORTC approach. Mean values are shown with 95% confidence interval. While there are clinically meaningful changes in the values over time in both arms, these are attributed to glioblastoma disease, its treatment, and progression. There is no worsening in the dose-enhanced arm that could be attributed to the intervention, such as headache or itchy skin. Here are the four domains of future, visual, motor and communication shown as well as symptoms. The higher the score the worse the domain and symptoms.


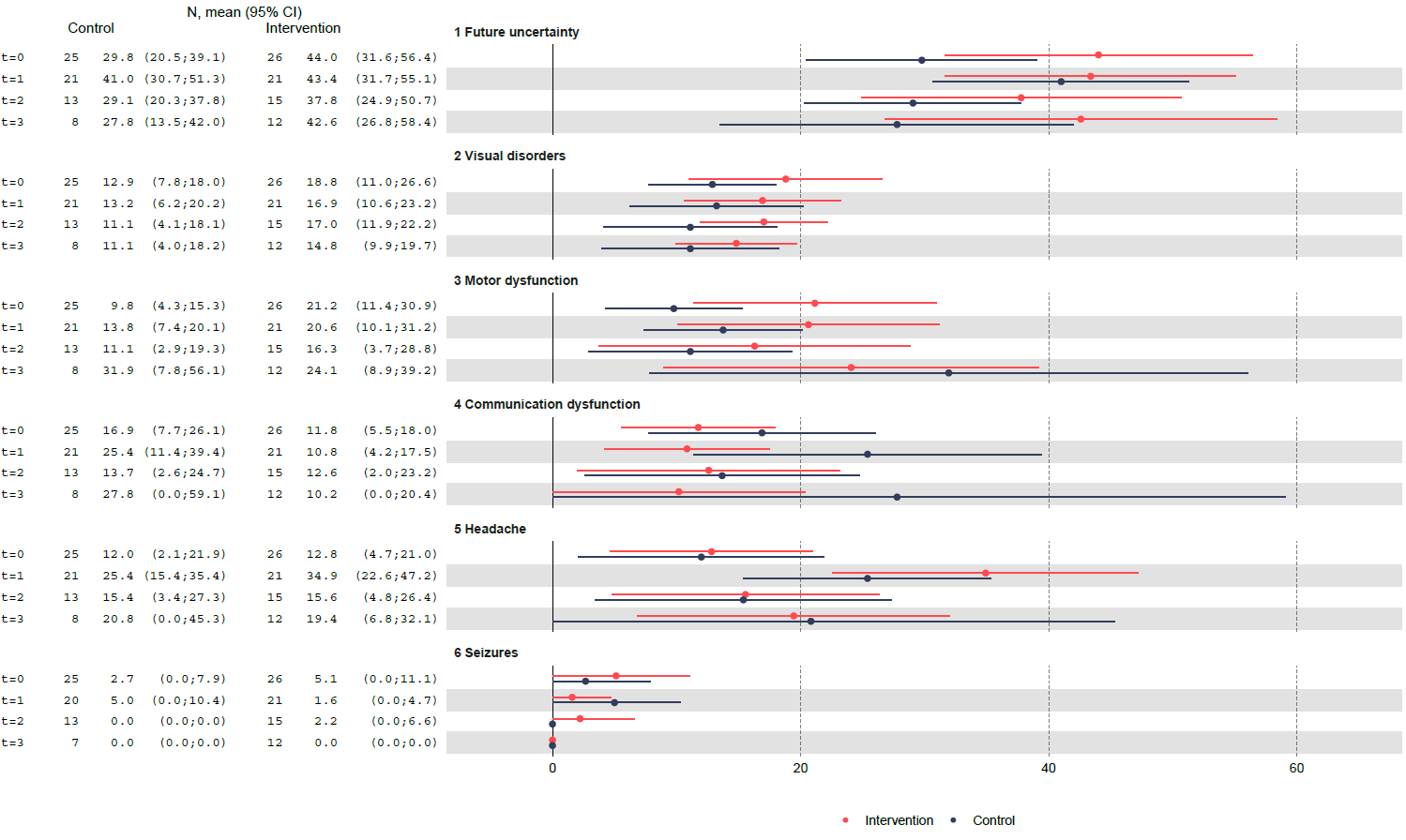


**Supplementary Figure 4. QLQ-BN20 for the intent-to-treat population (continued).**

T0 = Baseline, T1 = Period postsurgery but before initiating TTFields, T2 = 3-month control, T3 = 6-month control. The scores were calculated following the standardized recommended EORTC approach. Mean values are shown with 95% confidence interval. While there are clinically meaningful changes in the values over time in both arms, these are attributed to glioblastoma disease, its treatment, and progression. There is no worsening in the dose-enhanced arm that could be attributed to the intervention, such as headache or itchy skin. The higher the symptom score the worse the symptoms.
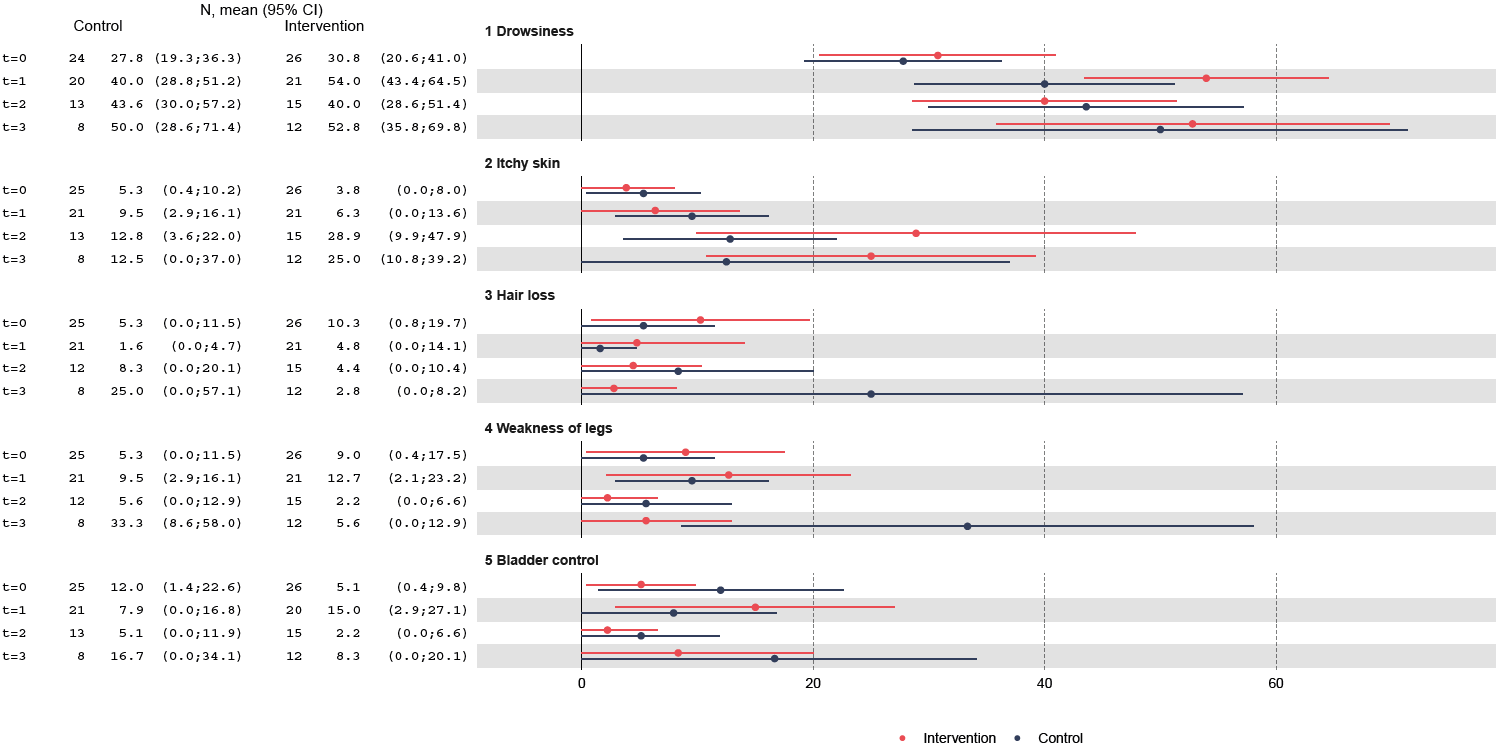


**Supplementary Figure 5. Prednisolone equivalent dose and KPS for the ITT population.**

T0 = Baseline, T1 = Period postsurgery but before initiation of TTFields therapy, T2 = 3-month control, T3 = 6-month control. Mean values are shown with 95% confidence interval. Both arms had a clinically meaningful reduction in steroid use after surgery, accompanied by a minor decline in KPS, which is expected. However, dose enhancement did not indicate less steroid use during the trial.


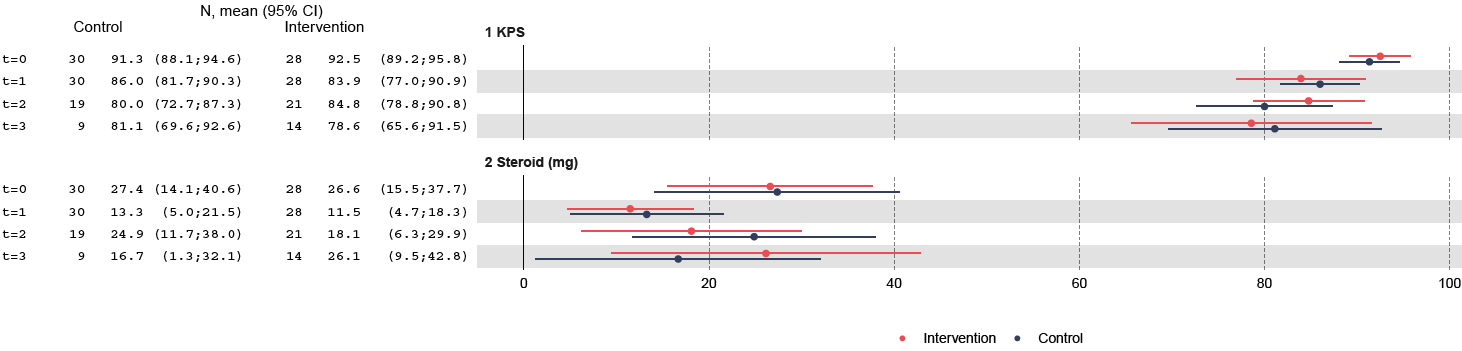

Supplement: vdaf245_Supplementary_Data [file vdaf245_supplementary_data.docx]
